# 1,1,3-Trimethyl-3-(4-nitro­phen­yl)indane

**DOI:** 10.1107/S1600536808027633

**Published:** 2008-10-25

**Authors:** Jian Men, Shi-Xu Yi, Fang Bo, Hua Chen, Guo-Wei Gao

**Affiliations:** a College of Chemistry, Sichuan University, Chengdu 610064, People’s Republic of China

## Abstract

In the title compound, C_18_H_19_NO_2_, the five-membered ring of the indane fragment adopts an envelope conformation, with the unsubstituted C atom, acting as the flap atom, deviating by 0.412 (3) Å from the plane through the remaining four atoms. The dihedral angle between the nitro­phenyl ring and the indane benzene ring is 72.5 (1)°. The distances from the two O atoms to the plane of the adjacent benzene ring are 0.113 (4) and 0.064 (4) Å.

## Related literature

For related literature, see: Bateman & Gordon (1976 ▶); Bezdek & Hrabak *et al.* (1979 ▶); Kumar *et al.* (1983 ▶); Men *et al.* (2008 ▶); Hanaineh-Abdelnour *et al.* (1999 ▶).
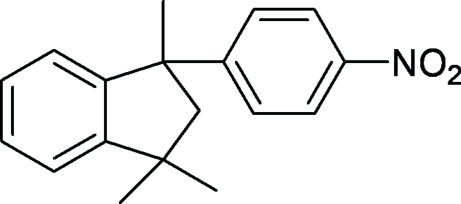


## Experimental

### 

#### Crystal data

C_18_H_19_NO_2_
*M* *_r_* = 281.34
Monoclinic, 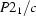

*a* = 11.305 (4) Å
*b* = 11.422 (2) Å
*c* = 11.963 (2) Å
β = 102.32 (4)°
*V* = 1509.1 (7) Å^3^
*Z* = 4
Mo *K*α radiation
μ = 0.08 mm^−1^
*T* = 294 (2) K
0.48 × 0.42 × 0.40 mm

#### Data collection

Enraf–Nonius CAD-4 diffractometer
Absorption correction: none
3836 measured reflections
2808 independent reflections
1569 reflections with *I* > 2σ(*I*)
*R*_int_ = 0.005
3 standard reflections every 200 reflections intensity decay: 0.3%

#### Refinement

*R*[*F* ^2^ > 2σ(*F* ^2^)] = 0.050
*wR*(*F* ^2^) = 0.148
*S* = 1.02
2808 reflections
197 parameters
H-atom parameters constrained
Δρ_max_ = 0.20 e Å^−3^
Δρ_min_ = −0.18 e Å^−3^

### 

Data collection: *DIFRAC* (Gabe & White, 1993 ▶); cell refinement: *DIFRAC*; data reduction: *NRCVAX* (Gabe *et al.*, 1989 ▶); program(s) used to solve structure: *SHELXS97* (Sheldrick, 2008 ▶); program(s) used to refine structure: *SHELXL97* (Sheldrick, 2008 ▶); molecular graphics: *ORTEP-3 for Windows* (Farrugia, 1997 ▶); software used to prepare material for publication: *SHELXL97*.

## Supplementary Material

Crystal structure: contains datablocks global, I. DOI: 10.1107/S1600536808027633/kj2096sup1.cif

Structure factors: contains datablocks I. DOI: 10.1107/S1600536808027633/kj2096Isup2.hkl

Additional supplementary materials:  crystallographic information; 3D view; checkCIF report

## Figures and Tables

**Fig. 1.**
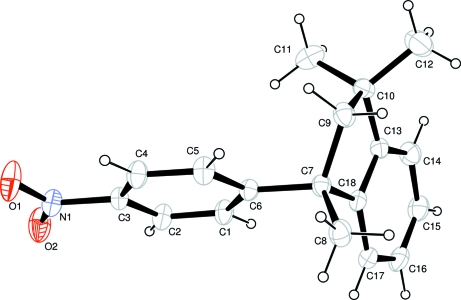
The molecular structure of (I), with displacement ellipsoids drawn at the 30% probability level.

## supplementary crystallographic information

### Comment

Maleimide and its substituted derivatives are well known monomers that have many applications in industry. It may be used to prepare heat resistant polymers or copolymers (Kumar *et al.*, 1983; Bezdek & Hrabak *et al.*, 1979). Excellent thermal properties of the maleimide polymers and copolymers have also attracted much attention (Hanaineh-Abdelnour *et al.*, 1999). The title compound is an important intermediate for synthesis of maleimide and its substituted derivatives. (Bateman & Gordon, 1976). Phenylindane amines is prepared by a process comprising acid-catalyzed dimerization of α-methylstyrene and subsequent nitration and reduction of the 1,1,3-trimethyl-3-phenyl-2,3-dihydro-1*H*-indene (Bateman & Gordon, 1976).

In the molecule of the title compound (Fig.1), the bond lengths and angles of the phenylidane moiety are comparable with those observed in 1,1,3,-trimethyl-3-phenyl-2,3-dihydro-1*H*-indene (Men *et al.*, 2008). Ring A (C1—C6) and B (C13—C18) are planar and have a dihedral angle of 72.5 (1)°. The B ring forms a dihedral angle of 27.1 (3)° with the plane defined by the indane Csp3 atoms C7, C9 and C10. The torsion angles O1—N1—C3—C2 and O2—N1—C3—C4 are -174.3 (2)° and -176.5 (2)°, respectively. The distances of the O atoms to the plane through the adjacent benzene ring are 0.113 (4) Å and 0.064 (4) Å, respectively. The five-membered ring of the indane fragment adopts an envelop conformation, with the unsubstituted C atom acting as the flag atom, deviating 0.412 (3) Å from the plane through the remaining four atoms.

### Experimental

To a three-necked, 250 ml flask equipped with a mechanical stirrer, 23.6 g (0.1 mol) 1,1,3-trimethyl-3-phenylindane, dissolved in 75 ml chloroform, was added. The flask was placed in an ice bath at 273 K. A previously mixed acidic solution containing 39.6 ml H_2_SO_4_ and 13.2 ml HNO_3_ was added dropwise to the phenylidane solution over 4 h at 273 K. The chloroform phase was isolated and washed with an aqueous bicarbonate solution and then with distilled water until neutral. The chloroform was removed with a rotovaporator, thereby yielding a viscous yellow liquid, which was recrystallized from methanol, afforded a light yellow powder (22.0 g, yield 78%, m.p.452–455 K). Single crystals suitable for X-ray diffraction were obtained at room temperature by slow evaporation of ethyl acetate over a period of several days.

### Refinement

H atoms were positioned geometrically (C—H = 0.93–0.97 Å) and refined with a riding model (*U* =0.06688–0.08804 Å^2^)

### Crystal data

**Table:**

C_18_H_19_NO_2_	*F*(000) = 600
*M**_r_* = 281.34	*D*_x_ = 1.238 Mg m^−^^3^
Monoclinic, *P*2_1_/*c*	Mo *K*α radiation, λ = 0.71073 Å
*a* = 11.305 (4) Å	Cell parameters from 25 reflections
*b* = 11.422 (2) Å	θ = 4.6–7.5°
*c* = 11.963 (2) Å	µ = 0.08 mm^−^^1^
β = 102.32 (4)°	*T* = 294 K
*V* = 1509.1 (7) Å^3^	Block, colourless
*Z* = 4	0.48 × 0.42 × 0.40 mm

### Data collection

**Table:**

Enraf–Nonius CAD-4 diffractometer	*R*_int_ = 0.005
Radiation source: fine-focus sealed tube	θ_max_ = 25.6°, θ_min_ = 1.8°
graphite	*h* = −13→13
ω/2θ scans	*k* = 0→13
3836 measured reflections	*l* = −9→14
2808 independent reflections	3 standard reflections every 200 reflections
1569 reflections with *I* > 2σ(*I*)	intensity decay: 0.3%

### Refinement

**Table:**

Refinement on *F*^2^	Secondary atom site location: difference Fourier map
Least-squares matrix: full	Hydrogen site location: inferred from neighbouring sites
*R*[*F*^2^ > 2σ(*F*^2^)] = 0.050	H-atom parameters constrained
*wR*(*F*^2^) = 0.148	*w* = 1/[σ^2^(*F*_o_^2^) + (0.0825*P*)^2^] where *P* = (*F*_o_^2^ + 2*F*_c_^2^)/3
*S* = 1.02	(Δ/σ)_max_ < 0.001
2808 reflections	Δρ_max_ = 0.20 e Å^−^^3^
197 parameters	Δρ_min_ = −0.18 e Å^−^^3^
0 restraints	Extinction correction: SHELXL97 (Sheldrick, 2008), Fc^*^=kFc[1+0.001xFc^2^λ^3^/sin(2θ)]^-1/4^
Primary atom site location: structure-invariant direct methods	Extinction coefficient: 0.017 (3)

### Special details

**Table:**

Geometry. All e.s.d.'s (except the e.s.d. in the dihedral angle between two l.s. planes) are estimated using the full covariance matrix. The cell e.s.d.'s are taken into account individually in the estimation of e.s.d.'s in distances, angles and torsion angles; correlations between e.s.d.'s in cell parameters are only used when they are defined by crystal symmetry. An approximate (isotropic) treatment of cell e.s.d.'s is used for estimating e.s.d.'s involving l.s. planes.
Refinement. Refinement of *F*^2^ against ALL reflections. The weighted *R*-factor *wR* and goodness of fit *S* are based on *F*^2^, conventional *R*-factors *R* are based on *F*, with *F* set to zero for negative *F*^2^. The threshold expression of *F*^2^ > σ(*F*^2^) is used only for calculating *R*-factors(gt) *etc*. and is not relevant to the choice of reflections for refinement. *R*-factors based on *F*^2^ are statistically about twice as large as those based on *F*, and *R*- factors based on ALL data will be even larger.

### Fractional atomic coordinates and isotropic or equivalent isotropic displacement parameters (Å^2^)

**Table:**

	*x*	*y*	*z*	*U*_iso_*/*U*_eq_	
O1	0.3433 (2)	−0.30042 (15)	0.44243 (16)	0.0958 (7)	
O2	0.3595 (2)	−0.17051 (16)	0.31987 (16)	0.0899 (7)	
N1	0.34612 (19)	−0.19883 (17)	0.41338 (17)	0.0587 (6)	
C1	0.3077 (2)	0.08995 (18)	0.53719 (17)	0.0510 (6)	
H1	0.3021	0.1679	0.5142	0.067 (3)*	
C2	0.3234 (2)	0.00613 (19)	0.45991 (18)	0.0525 (6)	
H2	0.3287	0.0263	0.3858	0.067 (3)*	
C3	0.3312 (2)	−0.10856 (17)	0.49490 (17)	0.0454 (5)	
C4	0.3226 (2)	−0.13981 (18)	0.60329 (18)	0.0523 (6)	
H4	0.3265	−0.2181	0.6251	0.067 (3)*	
C5	0.3082 (2)	−0.05412 (19)	0.67899 (17)	0.0521 (6)	
H5	0.3038	−0.0750	0.7531	0.067 (3)*	
C6	0.29992 (18)	0.06319 (18)	0.64821 (16)	0.0412 (5)	
C7	0.2858 (2)	0.15488 (17)	0.73769 (16)	0.0447 (5)	
C8	0.3994 (2)	0.1497 (2)	0.83509 (18)	0.0616 (7)	
H8A	0.4704	0.1586	0.8039	0.088 (3)*	
H8B	0.4022	0.0756	0.8735	0.088 (3)*	
H8C	0.3963	0.2117	0.8886	0.088 (3)*	
C9	0.1683 (2)	0.13740 (19)	0.78272 (18)	0.0544 (6)	
H9A	0.1440	0.0558	0.7758	0.068 (5)*	
H9B	0.1819	0.1594	0.8628	0.068 (5)*	
C10	0.0684 (2)	0.21467 (19)	0.71127 (19)	0.0546 (6)	
C11	−0.0054 (3)	0.1487 (2)	0.6080 (2)	0.0786 (8)	
H11A	−0.0597	0.2022	0.5606	0.088 (3)*	
H11B	−0.0513	0.0877	0.6342	0.088 (3)*	
H11C	0.0483	0.1151	0.5646	0.088 (3)*	
C12	−0.0166 (3)	0.2629 (3)	0.7825 (3)	0.0865 (9)	
H12A	0.0289	0.3071	0.8455	0.088 (3)*	
H12B	−0.0570	0.1992	0.8113	0.088 (3)*	
H12C	−0.0756	0.3127	0.7359	0.088 (3)*	
C13	0.1449 (2)	0.30897 (18)	0.67178 (18)	0.0479 (6)	
C14	0.1073 (3)	0.4174 (2)	0.6255 (2)	0.0638 (7)	
H14	0.0267	0.4399	0.6150	0.067 (3)*	
C15	0.1915 (3)	0.4909 (2)	0.5954 (2)	0.0656 (7)	
H15	0.1672	0.5637	0.5640	0.067 (3)*	
C16	0.3109 (3)	0.4589 (2)	0.6106 (2)	0.0629 (7)	
H16	0.3663	0.5101	0.5896	0.067 (3)*	
C17	0.3492 (2)	0.3515 (2)	0.65694 (19)	0.0539 (6)	
H17	0.4302	0.3298	0.6680	0.067 (3)*	
C18	0.2645 (2)	0.27631 (17)	0.68677 (16)	0.0437 (5)	

### Atomic displacement parameters (Å^2^)

**Table:**

	*U*^11^	*U*^22^	*U*^33^	*U*^12^	*U*^13^	*U*^23^
O1	0.170 (2)	0.0401 (10)	0.0774 (12)	0.0122 (11)	0.0259 (13)	−0.0062 (9)
O2	0.152 (2)	0.0637 (11)	0.0666 (12)	0.0012 (12)	0.0525 (12)	−0.0106 (9)
N1	0.0753 (15)	0.0474 (12)	0.0535 (12)	0.0026 (10)	0.0136 (10)	−0.0071 (9)
C1	0.0736 (18)	0.0365 (11)	0.0463 (11)	0.0024 (11)	0.0204 (11)	0.0039 (9)
C2	0.0739 (17)	0.0448 (13)	0.0426 (11)	−0.0010 (11)	0.0208 (11)	0.0024 (10)
C3	0.0503 (15)	0.0411 (11)	0.0457 (11)	0.0029 (10)	0.0124 (10)	−0.0051 (9)
C4	0.0687 (17)	0.0375 (12)	0.0505 (13)	0.0069 (11)	0.0124 (11)	0.0045 (9)
C5	0.0695 (17)	0.0468 (13)	0.0414 (11)	0.0073 (11)	0.0152 (11)	0.0075 (9)
C6	0.0439 (13)	0.0387 (11)	0.0424 (11)	0.0003 (10)	0.0126 (9)	0.0014 (9)
C7	0.0515 (14)	0.0420 (11)	0.0419 (11)	0.0028 (10)	0.0131 (10)	−0.0008 (9)
C8	0.0662 (17)	0.0681 (16)	0.0482 (12)	0.0069 (13)	0.0069 (11)	−0.0068 (11)
C9	0.0644 (17)	0.0517 (14)	0.0530 (12)	0.0029 (12)	0.0258 (11)	0.0020 (10)
C10	0.0490 (15)	0.0538 (14)	0.0648 (14)	−0.0004 (11)	0.0208 (12)	0.0004 (11)
C11	0.0667 (19)	0.0772 (19)	0.0885 (19)	−0.0178 (16)	0.0091 (15)	0.0037 (15)
C12	0.080 (2)	0.081 (2)	0.113 (2)	0.0119 (17)	0.0548 (19)	0.0061 (17)
C13	0.0481 (15)	0.0444 (12)	0.0532 (12)	0.0030 (11)	0.0152 (10)	−0.0057 (10)
C14	0.0608 (17)	0.0524 (15)	0.0776 (16)	0.0115 (13)	0.0135 (13)	0.0005 (12)
C15	0.083 (2)	0.0401 (13)	0.0751 (16)	0.0022 (14)	0.0209 (15)	0.0023 (11)
C16	0.082 (2)	0.0423 (14)	0.0683 (15)	−0.0165 (13)	0.0241 (14)	−0.0082 (11)
C17	0.0547 (16)	0.0492 (13)	0.0595 (13)	−0.0078 (12)	0.0160 (11)	−0.0112 (11)
C18	0.0497 (15)	0.0397 (12)	0.0439 (11)	−0.0010 (10)	0.0147 (10)	−0.0082 (9)

### Geometric parameters (Å, °)

**Table:**

O1—N1	1.214 (2)	C9—H9A	0.9700
O2—N1	1.205 (2)	C9—H9B	0.9700
N1—C3	1.454 (3)	C10—C12	1.518 (3)
C1—C2	1.368 (3)	C10—C13	1.518 (3)
C1—C6	1.384 (3)	C10—C11	1.533 (3)
C1—H1	0.9300	C11—H11A	0.9600
C2—C3	1.372 (3)	C11—H11B	0.9600
C2—H2	0.9300	C11—H11C	0.9600
C3—C4	1.368 (3)	C12—H12A	0.9600
C4—C5	1.366 (3)	C12—H12B	0.9600
C4—H4	0.9300	C12—H12C	0.9600
C5—C6	1.387 (3)	C13—C18	1.377 (3)
C5—H5	0.9300	C13—C14	1.385 (3)
C6—C7	1.530 (3)	C14—C15	1.374 (4)
C7—C18	1.513 (3)	C14—H14	0.9300
C7—C8	1.540 (3)	C15—C16	1.372 (3)
C7—C9	1.550 (3)	C15—H15	0.9300
C8—H8A	0.9600	C16—C17	1.377 (3)
C8—H8B	0.9600	C16—H16	0.9300
C8—H8C	0.9600	C17—C18	1.389 (3)
C9—C10	1.540 (3)	C17—H17	0.9300
			
O2—N1—O1	122.6 (2)	H9A—C9—H9B	108.4
O2—N1—C3	119.22 (19)	C12—C10—C13	112.8 (2)
O1—N1—C3	118.2 (2)	C12—C10—C11	109.2 (2)
C2—C1—C6	122.54 (19)	C13—C10—C11	110.26 (19)
C2—C1—H1	118.7	C12—C10—C9	111.9 (2)
C6—C1—H1	118.7	C13—C10—C9	100.42 (18)
C1—C2—C3	118.08 (19)	C11—C10—C9	112.0 (2)
C1—C2—H2	121.0	C10—C11—H11A	109.5
C3—C2—H2	121.0	C10—C11—H11B	109.5
C4—C3—C2	121.69 (19)	H11A—C11—H11B	109.5
C4—C3—N1	119.52 (19)	C10—C11—H11C	109.5
C2—C3—N1	118.78 (19)	H11A—C11—H11C	109.5
C5—C4—C3	118.9 (2)	H11B—C11—H11C	109.5
C5—C4—H4	120.5	C10—C12—H12A	109.5
C3—C4—H4	120.5	C10—C12—H12B	109.5
C4—C5—C6	121.78 (19)	H12A—C12—H12B	109.5
C4—C5—H5	119.1	C10—C12—H12C	109.5
C6—C5—H5	119.1	H12A—C12—H12C	109.5
C1—C6—C5	116.97 (18)	H12B—C12—H12C	109.5
C1—C6—C7	123.90 (18)	C18—C13—C14	120.2 (2)
C5—C6—C7	119.12 (17)	C18—C13—C10	112.06 (18)
C18—C7—C6	112.16 (15)	C14—C13—C10	127.8 (2)
C18—C7—C8	112.04 (18)	C15—C14—C13	118.7 (3)
C6—C7—C8	107.99 (17)	C15—C14—H14	120.6
C18—C7—C9	100.55 (17)	C13—C14—H14	120.6
C6—C7—C9	112.34 (17)	C16—C15—C14	121.3 (2)
C8—C7—C9	111.73 (18)	C16—C15—H15	119.4
C7—C8—H8A	109.5	C14—C15—H15	119.4
C7—C8—H8B	109.5	C15—C16—C17	120.4 (2)
H8A—C8—H8B	109.5	C15—C16—H16	119.8
C7—C8—H8C	109.5	C17—C16—H16	119.8
H8A—C8—H8C	109.5	C16—C17—C18	118.7 (2)
H8B—C8—H8C	109.5	C16—C17—H17	120.7
C10—C9—C7	108.33 (17)	C18—C17—H17	120.7
C10—C9—H9A	110.0	C13—C18—C17	120.7 (2)
C7—C9—H9A	110.0	C13—C18—C7	111.63 (19)
C10—C9—H9B	110.0	C17—C18—C7	127.7 (2)
C7—C9—H9B	110.0		
			
C6—C1—C2—C3	0.2 (4)	C7—C9—C10—C11	91.7 (2)
C1—C2—C3—C4	0.5 (4)	C12—C10—C13—C18	134.8 (2)
C1—C2—C3—N1	179.2 (2)	C11—C10—C13—C18	−102.8 (2)
O2—N1—C3—C4	−176.5 (2)	C9—C10—C13—C18	15.5 (2)
O1—N1—C3—C4	4.4 (3)	C12—C10—C13—C14	−45.1 (3)
O2—N1—C3—C2	4.9 (3)	C11—C10—C13—C14	77.3 (3)
O1—N1—C3—C2	−174.3 (2)	C9—C10—C13—C14	−164.4 (2)
C2—C3—C4—C5	−1.2 (4)	C18—C13—C14—C15	−0.1 (3)
N1—C3—C4—C5	−179.8 (2)	C10—C13—C14—C15	179.7 (2)
C3—C4—C5—C6	1.2 (3)	C13—C14—C15—C16	−0.2 (4)
C2—C1—C6—C5	−0.3 (3)	C14—C15—C16—C17	0.0 (4)
C2—C1—C6—C7	178.3 (2)	C15—C16—C17—C18	0.5 (3)
C4—C5—C6—C1	−0.4 (3)	C14—C13—C18—C17	0.6 (3)
C4—C5—C6—C7	−179.1 (2)	C10—C13—C18—C17	−179.25 (18)
C1—C6—C7—C18	7.7 (3)	C14—C13—C18—C7	−179.66 (18)
C5—C6—C7—C18	−173.75 (19)	C10—C13—C18—C7	0.4 (2)
C1—C6—C7—C8	−116.2 (2)	C16—C17—C18—C13	−0.8 (3)
C5—C6—C7—C8	62.3 (3)	C16—C17—C18—C7	179.52 (19)
C1—C6—C7—C9	120.1 (2)	C6—C7—C18—C13	103.5 (2)
C5—C6—C7—C9	−61.4 (3)	C8—C7—C18—C13	−134.8 (2)
C18—C7—C9—C10	25.7 (2)	C9—C7—C18—C13	−16.0 (2)
C6—C7—C9—C10	−93.8 (2)	C6—C7—C18—C17	−76.8 (3)
C8—C7—C9—C10	144.68 (18)	C8—C7—C18—C17	44.8 (3)
C7—C9—C10—C12	−145.3 (2)	C9—C7—C18—C17	163.6 (2)
C7—C9—C10—C13	−25.3 (2)		
